# DNA polymerases as chemotherapy targets: promise and challenges

**DOI:** 10.18632/oncotarget.26572

**Published:** 2019-01-18

**Authors:** Kristin A. Eckert, Ryan P. Barnes

**Affiliations:** Department of Pathology, Pennsylvania State University, The Jake Gittlen Laboratories for Cancer Research, Hershey, PA, USA

**Keywords:** polymerase eta, replication stress, TLS polymerase, ATR inhibitor

DNA replication stress is a key driver of genome instability and tumorigenesis, and is caused by intrinsic and environmental conditions that slow or stall DNA replication forks [1]. Sources of replication stress are abundant, and tumor cells have disarmed the cell cycle arrest and senescence mechanisms meant to protect genome stability and cell integrity, in favor of continued proliferation. In addition, tumor cells activate the ATR/Chk1 signaling axis to mitigate replication stress and avoid cell death. This complex signaling pathway regulates DNA replication, DNA repair, and cell cycle progression to allow tumor cell tolerance of replication stress, ultimately promoting tumor cell survival. Because replication stress can be caused by oncogene activation or by DNA damage caused by chemotherapy, inhibiting the ATR/Chk1 signaling pathway is a recent approach in clinical strategies [2]. Numerous clinical trials are in progress examining Chk1 inhibitors in solid tumors with oncogene amplification or tumor suppressor gene loss, and ATR inhibitors in combination with DNA damaging chemotherapeutics.

DNA polymerases are central players in the replication stress response [3]. In addition to the three canonical replicative DNA polymerases, human cells encode at least a dozen additional, specialized DNA polymerases to perform specific DNA synthesis functions during replication, repair and recombination. Exciting new research over the past few years has revealed that targeting DNA polymerases can have significant tumor cell killing effects under particular circumstances [4-6]. Experimentally, genetic approaches support the concept that DNA polymerases are therapeutic targets, paving the way for the clinical development of small molecule inhibitors.

**The Promise.** Sustained patient responses to chemotherapy can be low, and specialized polymerases that perform translesion synthesis (TLS) can promote tumor cell survival under conditions of genotoxic, therapy-induced replication stress [5]. For instance, DNA polymerase η (Pol η; *POLH* gene) is a key TLS polymerase mediating the bypass of cisplatin-induced DNA crosslinks, and increased POLH/Pol η expression is correlated with resistance to platinum-based chemotherapeutics [6]. Pol ζ is another specialized TLS polymerase that can be targeted to improve standard chemotherapy. Reducing expression of Pol ζ sensitizes tumor cells to cisplatin therapy in a mouse model, and Pol ζ is a synthetic lethal partner with combined ATR inhibition and cisplatin treatment [5].

We recently showed that Pol η regulation is central to the replication stress response, and discovered an unexpected link between Pol η and ATR that impacts tumor cell vulnerability to ATR/Chk1 targeted therapy [4]. Using multiple human cell lines, we showed that endogenous Pol η is upregulated at both the transcript and protein level, and relocalized to form intense nuclear foci in response to replication stress-inducing drugs that do not directly form DNA adducts. Importantly, this *POLH* transcriptional response is p53-independent, and the expression of other replication proteins did not change under the same treatments. Our data revealed a previously unknown mode of Pol η regulation during the replication stress response, and suggest that Pol η may be up-regulated early in tumorigenesis to mitigate the detrimental effects of replication stress.

We used Crisper/Cas9 to engineer Pol η-knockout (POLH^-/-^) derivatives, and showed that Pol η-deficient tumor cells have increased ATR/Chk1 activation, defective G2/M phase progression, and significantly reduced clonogenic survival following replication stress-inducing treatments [4]. ATR depletion together with replication stress dramatically elevated apoptotic signaling in Pol η-deficient cells, resulting in a 50–fold reduction in the clonogenic survival, a significantly greater response than wild–type cells. As a proof-of-principle experiment, we treated Pol η-deficient tumor cells with the highly selective ATR kinase inhibitor, VE-822, which has favorable outcomes in preclinical models. VE-822 treatment increased PARP-1 and Caspase-3 cleavage in Pol η-deficient cells, and inhibited the up-regulation of Pol η induced by replication stress [4]. These results suggest that targeting Pol η and ATR in combination may be a viable, new treatment strategy for cancer patients. Our synthetic lethality results suggest that the Pol η/POLH status of tumors should be evaluated to identify patients most likely to benefit from adjuvant therapy with ATR inhibitors. We hypothesize that low Pol η levels will sensitize tumor cells to ATR/Chk1 inhibitors. Conversely, our results indicate that high Pol η levels may confer resistance to ATR inhibitors. Using cBioPortal analyses, we showed that the *POLH* locus is primarily amplified in several cancers, including ovarian, melanoma and esophageal, and this amplification is correlated with increased mRNA expression [7].

**The Challenges.** The therapeutic efficacy of DNA polymerase inhibitors will be governed by the ability of inhibitors to selectively kill tumor cells without enhancing genome instability. One challenge will be to discover cellular contexts (e.g., specific genetic backgrounds or environments) in which tumor cells have an increased reliance on a particular DNA polymerase for continued survival and proliferation. However, a single polymerase can function in multiple genome maintenance pathways [3, 7], a fact that could increase toxicity to normal cells. A selective inhibitor of the replicative Pol δ has been developed which shows promise for homologous recombination-proficient tumors, possibly by inhibiting Pol δ functions in D-loop extension and double strand break repair [8]. Similarly, a small molecule Pol η inhibitor has been developed and shown to enhance tumor cell killing in response to cisplatin treatment [9]. Our results [4] also support the development of Pol η-specific inhibitors to use in an adjuvant setting with ATR/Chk1 inhibitors. Because Pol η plays key genome functions in addition to lesion bypass, including ALT telomere maintenance, homologous recombination, somatic hypermutation, and common fragile site stability [3], the long term effects of Pol η inhibition on normal cell toxicity must be carefully evaluated.

A second challenge for the use of DNA polymerase inhibitors is that cell survival is a strong selective pressure in the context of tumor therapy. Therefore, although a specific polymerase may be targeted, alternative, error-prone pathways exist in cells for completing DNA replication and repair [3]. Thus, shunting of DNA intermediates into error-prone pathways could fuel genome instability in tumor cells that survive treatment with specific polymerase inhibitors, limiting the sustained anti-tumor efficacy of such drugs.
